# Prevalence of HER-2 and Hormone Receptors and P53 Mutations in the Pathologic Specimens of Breast Cancer Patients

**DOI:** 10.1155/2014/564308

**Published:** 2014-11-12

**Authors:** Seyed Abbas Mirmalek, Maryam Hajilou, Seyed Alireza Salimi Tabatabaee, Yekta Parsa, Soheila Yadollah-Damavandi, Tina Parsa

**Affiliations:** ^1^Department of Surgery, Islamic Azad University, Tehran Medical Sciences Branch, Tehran, Iran; ^2^International Azad University of Kish, Kish, Iran; ^3^Students' Research Committee, Islamic Azad University, Tehran Medical Sciences Branch, Tehran, Iran

## Abstract

Prognostic factors are in interest for breast cancer as the second cause of malignancy deaths. Some have predictive values as human epidermal growth factor receptor-2 (HER-2) and estrogen receptor (ER). To access the incidence of HER2 and its relations to other factors, like age, pathology, ER, progesterone receptor (PR), and P53, 2000 pathologic blocks from 2750 total samples have been selected from 2011 to 2013 in Cancer Institute of Tehran. Incidence of HER2, ER, PR, and P53 was; 58.5%, 33.4%, 43.3%, and 65.4%, respectively. Invasive ductal carcinoma was the most pathologic type (82.2%) and 60%–70% positive HER2 and P53 had negative ER and PR (poor prognosis). The peak age of incidence of breast cancer was perimenopausal age group (46–55 years). Our cases had more positive HER2 and P53 and less positive PR and ER compared to other studies. High perimenopausal incidence as another finding assures the importance of breast cancer screening in these age groups.

## 1. Introduction

Among malignancies, breast cancer is one of the most common causes of mortality in women. Every woman is exposed to 8–10% of the risk of this disease during her life [1].

Several prognostic and predictive factors have been used for breast malignancies during last years. The most important factors include P53, progesterone and estrogen receptors, and human epidermal growth factor receptor-2 (HER-2). HER-2 gene or p185 is a part of the genetic code existing in all healthy individuals involved in the regulation of normal cell growth [2–4]. If extra copies of this gene are developed on the cell surface, the gene is overexpressed, making the cell tumoral. In general, the positive HER-2 receptor has the three following characteristics: rapid tumor growth, lower survival rate, and better response to adjuvant therapies such as chemotherapy and new drugs such as Herceptin [5–7]. Therefore, understanding the interaction between HER-2 polymorphism and breast cancer risk factors can be effective in determining treatment strategies and evaluating prognosis in this disease [8].

Increased HER-2 expression is involved in endometrial, stomach, and prostate cancers as well as breast cancer [9] but they show a lower prevalence of this gene polymorphism compared to breast cancer [10]. There are two methods for identification of the product of this gene: (1) immunohistochemistry (IHC) which shows cell surface proteins by staining with Ab and is more economical and (2) fluorescence in situ hybridization (FISH) that is more reliable and conducts the staining at the T-cell level. The role of estrogen in estrogen receptor- (ER-) positive cancer cells is the development of the cell cycle and prevention of apoptosis. Therefore, antiestrogens agents stop proliferation during the cell cycle. As a mitogen, estrogen can generally play the role of a prognostic factor in ER-positive cancer cells. In addition, it is of great value in determining the response to adjuvant therapies such as hormone therapy [7, 9–11]. The presence or absence of progesterone receptor (PR) can also be of importance for predicting response to hormonal treatments. Despite previous studies, simultaneous ER/PR positivity increases the response to endocrine therapies up to 75%. On the other hand, one-third of ER-positive and PR-negative cases responded to hormone therapies. A hypothesis suggests that the goal of the activity of ER is to facilitate the development of the tumor, so it is an effective aspect of this receptor, which is being investigated [11]. P53 is a transcription factor, which is proposed as a tumor suppressor. The mutation of this gene is associated with increased risk of breast cancer and poorer prognosis [12, 13]. The activity of P53 in tumor suppression via the stoppage of cell cycle or induction results in apoptosis. Many experiments have indicated that, besides their higher prevalence, p53 mutations are associated with drug resistance [14–17]. Currently, the staging evaluation that is used for determining the treatment process of patients is different from previous methods. Even grade I PR-negative and ER-negative patients need more aggressive adjuvant treatments and vice versa. Therefore, considering the importance of this matter, we decided to investigate the prevalence of the above factors in breast cancer patients and measure their relationship with each other and factors such as age and the type of pathology. With regard to the utmost importance of molecular biology in the diagnosis, treatment, and even prevention of cancer, the present research can be a basis for similar studies.

## 2. Materials and Methods

This retrospective cross-sectional study was performed on 2,000 women whose breast cancer pathology blocks were sent to the Cancer Institute of Imam Khomeini Hospital, Tehran, Iran, from 2011 to 2013. All cases whose pathology results were one of the types of breast malignancies and also were investigated for the above factors were selected from about 2,750 patients with breast lump whose pathology blocks were investigated from 2011 to 2013 in this center. The 2,000 present cases in this study were selected from the 2,750 patients. Patients were excluded if they had bilateral breast cancer, untreated brain metastases, osteoplastic bone metastases, pleural effusion, or ascites as the only evidence of disease, a second type of primary cancer. Patients were also excluded if they were pregnant or had received any type of therapeutic intervention since their malignancy was diagnosed. Afterwards, the test results of 2,000 of them were extracted from their pathology documents in the archive of the Cancer Institute and then recorded. All samples were evaluated by the IHC staining under the direct supervision of at least two pathology academics. HER-2 status was determined by means of IHC using the Dako HercepTest (Dako, Copenhagen, Denmark) and scored with the Dako scoring system [18]. Only patients who had weak-to-moderate staining of the entire tumor-cell membrane for Her-2 (referred to as a score of 2+) or more than moderate staining (referred to as a score of 3+) in more than 10 percent of tumor cells on IHC analysis were eligible for the study.

ER and PR receptors status was determined with a modified avidin-biotin (ABC) immunoperoxidase method according to standard protocols (Vector Laboratories, Burlingame, CA). The 3,3′-diaminobenzidine was used as the chromogen. The immunostaining results for ER and PR were assessed semiquantitatively and reported as positive if more than 5% of cells immunostained in a tumor. P53 overexpression was defined as more than 50% of the cells with strong nuclear staining [19].

Collected data are expressed as mean and standard deviation values. All data were analyzed by using ANOVA test with SPSS software (Version-13). *P* value < 0.05 was considered significant.

## 3. Results and Discussions

The highest rates of breast cancer were observed at the ages of 46–55, and lowest rates were observed under the age of 25 years and above 66 years. The most prevalent malignancy was invasive ductal carcinoma (IDC) at 82.2%. In this group, 33% and 56% were ER/PR-positive and ER/PR-negative, respectively. Ductal carcinoma in situ (DCIS) and mucinous carcinoma comprised the highest rates of HER-2-negative and HER-2-positive cases at 84.6% and 82.4%, respectively. Invasive tubular carcinoma (ITC) and medullary carcinoma reportedly comprised the highest rates of P53-negative and P53-positive cases at 85.7% and 90%, respectively (Table 1).

The highest rates of PR-negative and PR-positive cases were observed in medullary and invasive lobular carcinoma (ILC) and ITC at 56% and 85.7%, respectively.

The highest rates of positive HER-2 and negative HER-2 were observed at the ages of lower than 25 (76.9%) and 26–55 years (mean age range). The rate of HER-2-positive increased at the age of over 55 years.

The highest and lowest rates of ER-negative and ER-positive were observed at the ages of 56–65 and under 25 years at 75.5% and 61.5%, respectively. This significant difference with other studies can be due to sampling bias in the Cancer Institute. The highest rates of PR-negative and PR-positive were observed at the ages of over 66 years and under 25 years at 61.1% and 61.5%, respectively. There was no significant relationship between age and P53 (*P* = 0.295) (Table 2). It can be noted that 61.8% of ER-negative cases were positive regarding HER-2 (poor prognosis) and 48.1% of ER-positive cases were reported negative regarding HER-2 (good prognosis).

Additionally, 64.2% of PR-negative cases were positive regarding HER-2 (poor prognosis) and 49% of PR-positive cases were negative regarding HER-2 (good prognosis). Furthermore, 46.8% of P53-negative cases were negative regarding HER-2 (good prognosis) and 61.3% were positive regarding both factors (poor prognosis). Moreover, 84.4% the cases were negative regarding both factors and 98.7% of ER-positive cases were PR-positive.

In addition, 73.1% of ER-negative cases were P53-positive, 50.1% of ER-positive cases were P53-negative, 72.3% of ER-negative cases were P53-positive, and 43.8% of ER-positive cases were P53-negative. Interestingly, the result obtained regarding the relationship between HER-2 and the type of malignancy and ER and PR had a relationship with the similarity of ER and PR receptors in a way that there was a significant relationship between the above 3 factors only if both ER and PR receptors were reported to be positive or negative. Regarding the relationship between age and the HER-2 receptor, poorer prognosis was associated with younger ages as expected (with more positive HER-2). However, in the others hormone receptor-positive (ER and PR), patients have a higher age range and a good prognosis. This point indicates the need for further research with regard to the lower prevalence of ER and PR in our study.

## Figures and Tables

**Table 1:** Frequency of hormone receptors and breast cancer types.

Breast cancer types	Receptors types	Percent
IDC	ER/PR+	33%
	ER/PR−	56%
DCIS, mucinous carcinoma	HER-2+	82.4%
	HER-2−	84.6%
IDC, medullary carcinoma	P53+	90%
	P53−	85.7%
medullary carcinoma, ILC, and IDC	PR+	85.7%
	PR−	56%

**Table 2:** Highest and lowest frequency of HER-2, ER, and PR status within different age groups.

Receptors status	Highest age range (%)	Lowest age range (%)
HER-2+/−	<25 y/o (76.9%)	26–55 y/o
ER+/−	56–65 y/o (75.5%)	<25 y/o (61.5%)
PR+/−	>66 y/o (61.1%)	<25 y/o (61.5%)
